# The Role of HSPB8, a Component of the Chaperone-Assisted Selective Autophagy Machinery, in Cancer

**DOI:** 10.3390/cells10020335

**Published:** 2021-02-05

**Authors:** Riccardo Cristofani, Margherita Piccolella, Valeria Crippa, Barbara Tedesco, Marina Montagnani Marelli, Angelo Poletti, Roberta M. Moretti

**Affiliations:** Dipartimento di Scienze Farmacologiche e Biomolecolari (DiSFeB), Dipartimento di Eccellenza 2018–2022, Università degli Studi di Milano, 20133 Milan, Italy; riccardo.cristofani@unimi.it (R.C.); margherita.piccolella@unimi.it (M.P.); valeria.crippa@unimi.it (V.C.); barbara.tedesco@unimi.it (B.T.); marina.marellimontagnani@unimi.it (M.M.M.); roberta.moretti@unimi.it (R.M.M.)

**Keywords:** PQC, chaperones, HSPB8, autophagy, CASA, cancer

## Abstract

The cellular response to cancer-induced stress is one of the major aspects regulating cancer development and progression. The Heat Shock Protein B8 (HSPB8) is a small chaperone involved in chaperone-assisted selective autophagy (CASA). CASA promotes the selective degradation of proteins to counteract cell stress such as tumor-induced stress. HSPB8 is also involved in (i) the cell division machinery regulating chromosome segregation and cell cycle arrest in the G0/G1 phase and (ii) inflammation regulating dendritic cell maturation and cytokine production. HSPB8 expression and role are tumor-specific, showing a dual and opposite role. Interestingly, HSPB8 may be involved in the acquisition of chemoresistance to drugs. Despite the fact the mechanisms of HSPB8-mediated CASA activation in tumors need further studies, HSPB8 could represent an important factor in cancer induction and progression and it may be a potential target for anticancer treatment in specific types of cancer. In this review, we will discuss the molecular mechanism underlying HSPB8 roles in normal and cancer conditions. The basic mechanisms involved in anti- and pro-tumoral activities of HSPB8 are deeply discussed together with the pathways that modulate HSPB8 expression, in order to outline molecules with a beneficial effect for cancer cell growth, migration, and death.

## 1. Introduction

Human cells have different fine-tuned systems that act as defense mechanisms against a large number of different environmental stresses. Among these, one identified in the 1960s by Ferruccio Ritossa is the heat shock response (HSR), an extraordinary mechanism shared by procaryotes and eukaryotes which responds to and counteracts several potentially harmful cell stresses. In fact, the HSR is now identified more generally as a stress response, being triggered in different ways by a variety of stressful conditions affecting the cells. The stress response may arise outside (i.e., heat shock) or inside (i.e., protein misfolding) the cells, and it is based on the interplay between the nucleus and organelles such as mitochondria, the endoplasmic reticulum, autophagosomes, and lysosomes as well as membrane-less organelles such as P bodies and stress granules (SGs). The stress response is based on several cellular processes such as gene expression, protein synthesis, and protein and organelle degradation and it is involved in aging and in many human diseases such as neurodegeneration, inflammation, obesity, diabetes, autoimmune diseases, atherosclerosis, and cancer [1,2].

The stress response, including the HSR, is based on a rapid and transient gene expression mechanism that controls the expression of molecular chaperones: the heat shock proteins (HSPs). The six molecular chaperone families are ubiquitous and broadly conserved; even if not all of them are induced by heat shock, they are, indeed, differentially controlled by different stresses. Chaperones were originally grouped based on their apparent molecular mass, while now, they are classified mainly by their functions in the folding processes (HSP110s, HSP90s, HSP70s, HSP60s, HSP40s, and small heat shock proteins (sHSPs)), but there are similarities between the two nomenclatures; in fact, based on the HUGO Gene Nomenclature Committee, the new classification recently adopted is as follows: HSPB (sHSP), DNAJ (HSP40), HSPD (HSP60), HSPA (HSP70), HSPC (HSP90), and HSPH (HSP110) [3], and reflects both the functions and the sizes of the members of each subfamily.

In this review, we will focus on the human HSPB8, a member of the HSPBs, which are a ubiquitous family of ATP-independent stress proteins, whose activity is mediated by other ATP-dependent chaperones (i.e., HSPAs), defining whether HSPBs clients can be refolded or degraded [4,5]. HSPBs are able to bind unfolded/misfolded substrates and subsequently new and larger oligomers are formed, preventing irreversible client aggregation and allowing ATP-dependent chaperones recognition to assist protein refolding [6]. HSPBs contribute to protein quality control (PQC) and work to prevent protein aggregation and to generate a pool of non-native proteins that can be rapidly folded [7]. The human genome encodes 10 HSPBs named HSPB1 through HSPB10, with an apparent molecular mass of 12–43 kDa [8]. Despite their name, the expression of HSPBs can be dependently or independently regulated by heat shock transcription factors (HSFs). In fact, it relies on the combinatory effects of many transcription factors. The basal expression or stress inducibility of HSPBs is therefore regulated by different cis-elements localized in the HSPB regulatory regions [9].

A common key feature of HSPBs is the alpha-crystallin domain (ACD), which refers to the best-known family member, the eye lens protein alpha-crystallin [10]. The ACD is flanked by a variable N-terminal domain (NTD) and a short C-terminal domain (CTD), which show high heterogeneity both in sequence and size among the HSPBs. All the three domains are involved in determining the quaternary structure adopted by the HSPBs. Indeed, while the ACD mediates the dimerization of HSPBs, the ACD flanking regions affect HSPBs oligomerization. Since the NTD and CTD highly differ among the HSPBs, the size and composition of HSPB oligomers can vary, ranging from mainly dimers to 600 kDa hetero-oligomers [11,12,13]. A dynamic association/dissociation has been suggested as a primary regulator of HSPB functions and it is often induced by protein phosphorylation [14].

HSPB8, also named small stress protein-like protein (sHSP22), protein kinase H11(H11), E2-induced gene 1 protein (E2IG1), or alpha-crystallin C chain (CRYAC), is different from other HSPBs, since it is preferentially found as monomers and homodimers, even if the protein can also interact with other HSPBs forming heterodimers [15,16,17]. In addition, like HSPB5 and HSPB6, HSPB8 represents an “atypical” member of the HSPB family because in mammalian cells, it can form stable complexes with the Bcl2-associated athanogene 3 (BAG3), which is thought to be the obligate partner of HSPB8 [18,19].

## 2. Tissue Distribution of HSPB8

In basal conditions, the heart and the skeletal muscle are the two most noteworthy tissues in which HSPB8 is expressed at relatively high levels. However, these tissues also express many other HSPBs, such as HSPB1, HSPB2, HSPB3, HSPB5, HSPB6, and HSPB7, possibly because during life, muscle tissues are subjected to several stressful conditions (including prolonged mechanical stress, hypoxia, damaged protein exposure). HSPB8 is also highly expressed in the brain, where it can be found both in neurons and in glial cells. Neuronal HSPB8 expression appears to be particularly relevant as a neuroprotective mechanism; for example, under stressful conditions, such as proteotoxic stresses, motoneurons express a very high level of HSPB8 in an attempt to protect themselves from damage [20]. Lower HSPB8 expression has been observed in the prostate, placenta, lungs, kidneys, and skin, while the ovaries, testes, liver, pancreas, and spleen appear to be devoid of HSPB8 [21]. As with many other HSPs, HSPB8 expression can be triggered by a variety of stresses, including starvation, proteasome inhibition, misfolded proteins accumulation, nuclear factor-Kappa B (NF-κB) activation, autophagy activation, microtubule destabilization, and occasionally heat shock [20,22,23,24,25,26].

## 3. Structure of HSPB8

**Figure 1:**
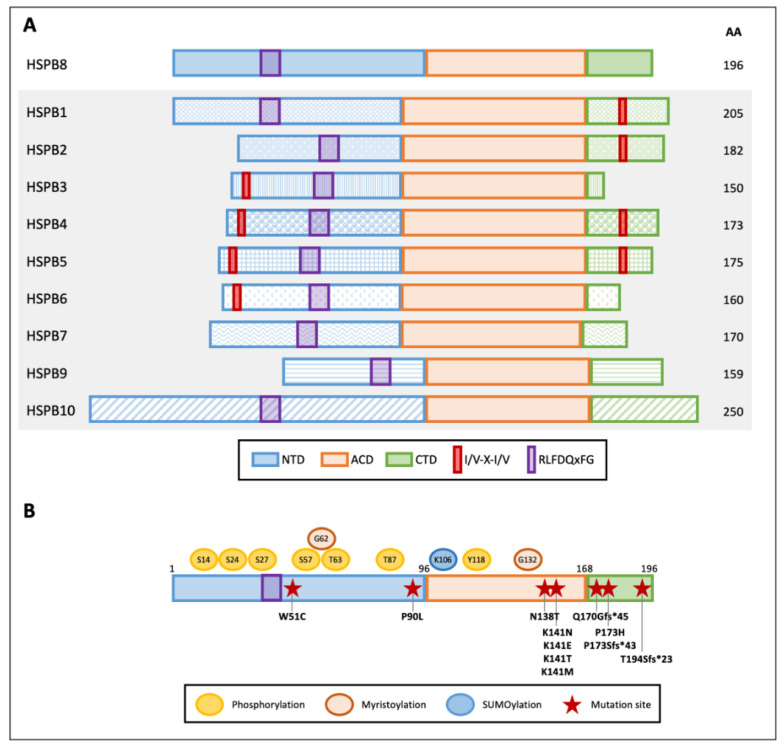
Graphical representation of human small heat shock proteins (HSPBs) structure and features. (**A**) Schematic diagram of the domains within human HSPBs. NTD and CTD indicate the variable N- and C-terminal domains, respectively; ACD indicates the conserved alpha-crystallin domain. Red boxes and purple boxes indicate the I/V-X-I/V motifs and the RLFDQxFG conserved sequence, respectively. Amino acid length is indicated on the right. (**B**) HSPB8 schematic structure, post-translational modifications, and mutations. Post-translational modification residues are reported: phosphorylation sites are indicated in yellow, predicted myristoylation sites in orange, and a SUMOylation site in light blue. Red stars indicate HSPB8 mutations reported in the literature.

HSPB8 (Figure 1A) is composed of 196 amino acids with an apparent molecular mass of 21.6 kDa. Its ACD is located close to the CTD approximately between amino acids 86 and 176. The short CTD interacts with aberrantly exposed hydrophobic regions of the substrate, preventing its aggregation during chaperone activity. Different from other members of the HSPB family (HSPB1, HSPB2, HSPB4, and HSPB5), HSPB8 does not contain the conserved I/V-X-I/V motif responsible for oligomeric assembly [27,28]. Indeed, this motif interacts with the neighboring ACD in a pocket between strands β4 and β8, acting as a bridge between the dimers. Instead, a hydrophobic pocket mediates the HSPB8 binding to two I/V-X-I/V domains, namely, IPV domains, present in the BAG3 protein [29], thus explaining the preferential binding of HSPB8 to BAG3 rather than HSPB8 assembly into large oligomers. In this interaction, two molecules of HSPB8 complement one BAG3 molecule, forming a functional chaperone complex (see below).

The NTD is an intrinsically disordered region enriched in Pro and Arg residues, which is highly susceptible to proteolysis [30,31]. The NTD contains the conserved RLFDQxFG motif that was shown to play an essential role in the interaction with other HSPs [32].

HSPBs can undergo post-translational modifications (Figure 1B). HSPB8 has several putative phosphorylation sites, but only Ser-14, Ser-24, Ser-27, Ser-57, Thr-63, Thr-87, and Tyr-118 phosphorylation has been described so far [33,34]. Ser-14 and Thr-63 phosphorylation was found to be mediated by protein kinase C (PKC), while p44 mitogen-activated protein kinase phosphorylates Ser-27 and Thr-87 [33,35]. Moreover, Ser-24, Ser-27, and Thr-87 are potential sites of ERK1 phosphorylation. Phosphorylation of Ser-24, Ser-27, and Thr-87 was associated with an increase in HSPB8 dimerization and a reduction in its degradation. Instead, phosphorylation of Ser-24 and Ser-27 decreases HSPB8 chaperone activity, while phosphorylation of Thr-87 increases HSPB8 chaperone activity [36]. A similar activity has been linked to Ser-57 phosphorylation mediated by cyclic AMP-dependent protein kinase (PKA) [37]. While HSPB8 phosphorylation has been evaluated through in vitro assays, its role in disease has not been elucidated.

On the other hand, HSPB8 SUMOylation at Lys-106 in MCF-7 cancer cells has been recently described. Noteworthy, HSPB8 SUMOylation is promoted by HSPB1-mediated recruitment of SUMO-2/3 enzymes. This leads to an increase in HSPB8 expression, which favors breast cancer progression (see below) [38].

Despite the fact that it is unknown whether HSPB8 is myristoylated, and that its functional significance is unknown, HSPB8 has two predicted myristoylation sites at residues Gly-62 and Gly-132 that may enhance its membrane-binding potential [39].

## 4. Disease-Associated Mutations of HSPB8

Few mutations have been described in the HSPB8 gene and are associated with disease (Figure 1B). The first identified mutation in the HSPB8 gene in cancer cells causes a W51C substitution in the protein sequence and was reported in one melanoma line with an important transforming potential. The mutant W51C HSPB8 acquires a predicted different secondary structure characterized by seven additional β-turns [40] and, in this way, is capable of altering HSPB8 protein–protein interaction, conferring a dominant anti-apoptotic activity to the mutated protein [40]; this greatly differs from the typical pro-apoptotic activity of wtHSPB8 in melanoma cells. Another HSPB8 anti-apoptotic mutation is the missense mutation P173H, found in human melanoma cell line MeWo. P173H HSPB8 loses its propensity to bind the transforming growth factor-beta-activated kinase 1 (TAK1), an interaction required to induce apoptosis [41], and it is thought to confer 5-Aza-deoxy-cytidine resistance to these melanoma cells.

The majority of HSPB8 mutations have been identified in patients with Charcot–Marie–Tooth (CMT) type 2L [42,43], myopathy, and distal hereditary motor neuropathy (dHMN) type IIa [44,45,46]. In particular, mutations of the conserved lysine at position 141 (K141N, K141E, K141T, and K141M), which are the most frequently described, affect HSPB8 dimerization and BAG3 interaction, causing HSPB8 toxic aggregation. Two other new HSPB8 mutations that have been identified in dHMN patients are P90L and N138T, but their role in disease needs further characterization [47]. Instead, the recently described frame shift mutation pP173Sfs*43, caused by the duplication c.515dupC in the *HSPB8* gene, has been associated with autosomal dominant rimmed vacuolar myopathy. Moreover, the deletion c.508–509delCA (pQ170Gfs*45) and the duplication c.577–580dupGTCA (pT194Sfs*23) in the *HSPB8* gene have been described as responsible for adult-onset axial and distal myopathy and proximal limb-girdle rimmed vacuolar myopathy, respectively [48,49]. These mutations are predicted to be translated in HSPB8 variants with an elongated C-terminal domain [46]. However, since no elongated HSPB8 has been detected in samples derived from patients, due to mRNA decay or protein instability, a haploinsufficiency mechanism has been hypothesized to explain the disease-related phenotype [46,48].

## 5. Role of HSPB8 in PQC

HSPB8 plays an important role in the degradative pathways, although it has also been reported to directly promote the re-folding of specific substrates, such as the HSPB5 R120G mutant protein [50,51]. The two major degradative pathways are (i) the ubiquitin–proteasome system (UPS) and (ii) the lysosomal-mediated system. UPS is the primary system involved in the degradation of ubiquitinated proteins and it is characterized by a relatively low capacity but also by a high selectivity for monomeric proteins. The lysosomal-mediated system is typically divided into: (i) microautophagy, (ii) chaperone-mediated autophagy (CMA), and (iii) macroautophagy (hereafter referred to autophagy). This system has high capacity but was generally considered to be characterized by a low selectivity for substrates, and, indeed, it is capable of degrading large protein aggregates and damaged organelles, in a process known as “in bulk” autophagy; however, it is now clear that several forms of autophagy (in particular CMA, chaperone-assisted selective autophagy (CASA), microautophagy, and autophagy of specific organelles, including aggrephagy) are highly selective and regulated by several pro-autophagic factors [52,53,54]. All these pathways are regulated by specific chaperones and co-chaperones which determine proteins or organelles fate. In the case of protein degradation, the chaperones and co-chaperones route substrates either to the UPS or to autophagy, preventing an imbalance of these two degradative pathways that may have deleterious effects, which could become responsible for several human diseases [55,56,57,58,59,60,61].

**Figure 2:**
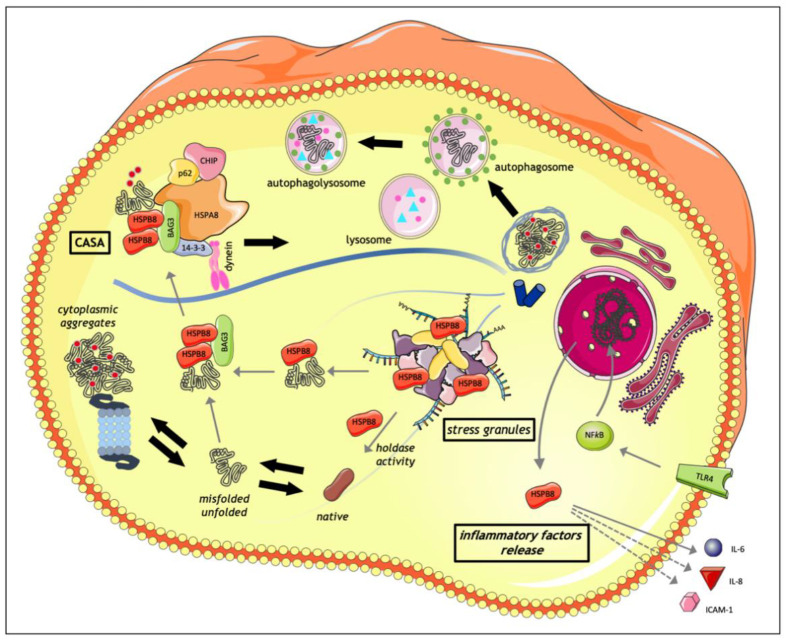
Schematic representation of HSPB8 activities in the interphase. HSPB8 is mainly involved in the maintenance of intracellular proteostasis, being an important component of the protein quality control system (PQC). In this context, HSPB8, together with its partner BAG3, HSPA8, and the STUB1/CHIP proteins, forms the chaperone-assisted selective autophagy (CASA) complex, which assists the degradation of aberrantly folded substrates via autophagy. The CASA complex is able to recognize and bind several misfolded proteins and it drives them along microtubules to the microtubule organization center (MTOC) for their insertion into autophagosome and subsequent lysosomal degradation. Moreover, HSPB8 is directly involved in the maintenance of stress granule (SG) dynamism: in fact, in stress conditions, HSPB8 is upregulated and it is specifically recruited to SGs where it both assists the folding of defective ribosomal products (DRiPs) (holdase activity) and promotes the extraction of misfolded substrates from SGs, for their following BAG3-HSPA-mediated degradation. Another role of HSPB8 is in the inflammatory response. HSPB8 can induce the release of inflammatory factors (IL-6, IL-8, ICAM1) from human smooth muscle cells and brain pericytes and astrocytes in models of cerebral amyloid angiopathy. It is also involved in TLR4-mediated dendritic cell maturation and activation accompanied by IL-6 production. This figure was created using Servier Medical Art templates, which are licensed under a Creative Commons Attribution 3.0 Unported License; https://smart.servier.com (accessed on 27 January 2021).

HSPB8 is a key component of CASA (Figure 2), an autophagic pathway distinct from the previously listed CMA. In fact, CMA mediates protein degradation through a protein direct translocation across the lysosomal membrane and requires specific consensus motifs in the target proteins which are recognized by a selective HSPA and LAMP2A. Instead, HSPB8 directly interacts with BAG3, HSPA8, and the carboxyl-terminus of HSC70-interacting protein STUB1/CHIP, forming the CASA complex. Based on the interaction between BAG3 and the dynein motor complex, the CASA complex is then routed to the microtubule organization center (MTOC), where autophagosome nucleation occurs [26,62,63,64]. Substrates engulfment into autophagosomes requires their STUB1/CHIP-mediated ubiquitination and their recognition by the autophagic adaptor SQSTM1/p62 (p62). Therefore, the CASA complex is responsible for the recognition of misfolded proteins and facilitates their degradation through the autophagosome–lysosome pathway [65]. This proteostasis mechanism is essential for protein assembly and turnover in striatal skeletal muscles and the heart, which are subjected to mechanical, thermal, and oxidative stress during contraction. Besides the role of HSPB8 in muscle maintenance, HSPB8 activity has also been described in neuronal cells. Indeed, HSPB8 facilitates the degradation of several misfolded proteins (Htt43Q, Androgen Receptors 46Q and 112Q, mutated SOD1, mutated TDP-43, and dipeptide repeat proteins from the C9ORF72 gene containing the expanded G_4_C_2_ stretch) responsible for neurodegenerative diseases, such as frontotemporal dementia (FTD), amyotrophic lateral sclerosis (ALS), spinal and bulbar muscular atrophy (SBMA), and Huntington’s disease (HD), and contributes to maintain a normal autophagic flux and to promote neuronal survival [20,23,26,60,66]. Interestingly, the HSPB8 capability to recognize not only misfolded proteins but also aberrant peptides such as dipeptide repeat proteins suggests that HSPB8 may recognize a large variety of aberrant polypeptides and misfolded proteins that may aggregate in cells.

The HSPB8-BAG3-HSPA complex is also involved in the maintenance of SG dynamism (Figure 2) [67]. Stress conditions trigger the HSPB8 dissociation from the BAG3-HSPA complex, allowing its recruitment into SGs where, thanks to its holdase activity, HSPB8 assists the folding of misfolded proteins and defective ribosomal products (DRiPs), preventing their irreversible aggregation and the conversion of SGs into stable forms. Only later HSPB8 recruits BAG3 to promote the misfolded proteins processing by the HSPA machinery [67]. Moreover, HSPB8 interacts with other RNA-binding proteins: (i) the DEAD box protein Ddx20 (gemin3, DP103) and (ii) Src-associated in mitosis of 68 kDa (Sam68), involved in RNA functions and processing such as pre-mRNA processing, RNA turnover, RNA transcription, and RNA export [68,69]. Interestingly, BAG3-independent activity of HSPB8 is also supported by the fact that the induction of HSPB8 expression by proteasome inhibition occurs even in BAG3-silenced cells. Indeed, in the early response to stress, HSPB8 promotes the nucleation of small ubiquitinated aggregates and p62 recruitment, upstream of BAG3 intervention. Then, HSPB8 promotes the coupling of p62- and ubiquitin-positive small aggregates to the BAG3 machinery, allowing subsequent misfolded proteins degradation [20,60,70].

Despite all these functions, HSPB8 does not appear to be fundamental for cell survival, since the generation of HSPB8 knock-out mice did not result in an apparent phenotype and the mice appeared to be normal [71]. It is possible that specific compensatory mechanisms may prevent the lack of HSPB8 activity in mediating the degradation of aberrant proteins. Alternatively, it is possible that the lack of HSPB8 activity may become evident only under stressful conditions (as it may be expected from its transcriptional control), but unfortunately, no data on HSPB8 (KO) mice exposed to stressors have been reported so far. Notably, abnormal accumulation of autophagosomes unable to fuse with lysosomes has been observed in cellular and mouse models overexpressing the HSPB8 K141E mutation compared to wild-type HSPB8 models. These results suggest that HSPB8 is also involved in the autophagosome maturation process contributing to finalize the autophagy flux [71,72]. Moreover, HSPB8 increases the selective removal of irreversible damaged proteins after heat shock, through HSPB8 and BAG3 upregulation induced by activation of NF-κB, increasing cell survival in hyperthermia conditions [73].

## 6. Role of HSPB8 in Cell Division Machinery

**Figure 3:**
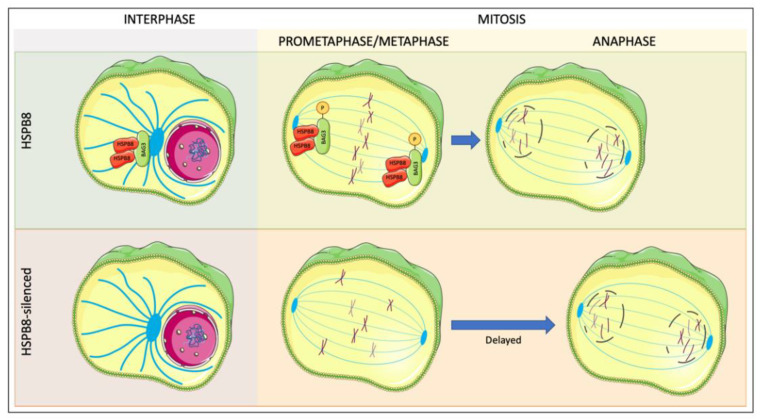
HSPB8 and BAG3 play an important role in mitosis. Before nuclear envelope breakdown, HSPB8 and BAG3 localize together at centrosome. With the beginning of prometaphase and the nuclear disassembly, BAG3 becomes hyperphosphorylated, and at the metaphase to anaphase transition, it re-localizes at the spindle poles participating in the formation of the correct mitotic spindle orientation. BAG3 behavior is closely dependent on HSPB8, and a reduction in BAG3 hyperphosphorylation occurs in HSPB8-silenced mitotic cells. BAG3 silencing increases the number of cells arrested at prometaphase, or at metaphase with unaligned chromosomes at the spindle poles. HSPB8 silencing also causes a delay and an alteration in cytokinesis. This figure was created using Servier Medical Art templates, which are licensed under a Creative Commons Attribution 3.0 Unported License; https://smart.servier.com (accessed on 27 January 2021).

Among the noncanonical functions of the HSPBs, their interactions with cytoskeleton elements have been identified. For example, the HSPB8-BAG3 complex is a key element of cytoskeletal proteostasis in muscle cells [74], but little is known about the function of this chaperone complex in dividing cells. Cell division is a refined process that, among other processes, requires close collaboration between actin fibers and the mitotic spindle responsible for chromosome segregation [75]. Recently, it has been shown that HSPB8 and BAG3 play an important role during mitosis in mammal cells, and that this process also involves the autophagic receptor p62 [76]. Indeed, in HeLa cells, HSPB8, BAG3, and p62 collaborate in the progression of mitosis, controlling the proper segregation of chromosomes in the two daughter cells. The joint action of these three proteins also influences the orientation of the mitotic spindle and the assembly of particular actin structures that appear only during mitosis. One of the most interesting observations concerns the intracellular co-localization of these proteins, especially in relation to the mitotic machinery (Figure 3). Before nuclear envelope breakdown, HSPB8 and BAG3 localize together at centrosomes with the centrosomal proteins beta-tubulin and CEP170. With the beginning of prometaphase and the nuclear disassembly, BAG3 becomes hyperphosphorylated, and at the metaphase to anaphase transition, it re-localizes at the spindle poles participating in the formation of the correct mitotic spindle orientation. This behavior of BAG3 is closely dependent on HSPB8, and a reduction in BAG3 hyperphosphorylation occurs in HSPB8-silenced mitotic cells. Therefore, the specific localizations of BAG3 at these different stages during the M phase indicate that the HSPB8-BAG3 complex plays an important role in mitosis. This is supported by the increased number of cells arrested at prometaphase, or at metaphase with unaligned chromosomes at the spindle poles in BAG3-silenced cells. Moreover, the incidence of aberrant mitosis and cytokinesis is increased in the cells able to reach anaphase. In agreement with these observations on BAG3 silencing, in the MCF-7 breast cancer (BC) cell line, HSPB8 silencing causes a block of the cell cycle in the G0/G1 phase and a decrease in cell proliferation, confirming that HSPB8 is involved in the mechanisms that regulate the cell cycle and cell proliferation [77]. From the mechanistic perspective, in this process, the mitotic actin-based cytoskeleton undergoes wide remodeling during mitosis to allow changes in the cell shape and interacts with the spindle for its correct alignment [78]. Moreover, the formation of a rigid actin cortex allows not only the cell–substrate interaction, but also the interaction with astral microtubules [75].

The CASA complex, or at least the two major components of this complex (HSPB8 and BAG3, together with p62), could participate in the proper assembly of actin-based structures that control spindle positioning. In fact, the silencing of BAG3, HSPB8, or p62 leads to a marked disorganization of the actin dots surrounding the ventral cortex of round mitotic cells [76]. Therefore, the HSPB8-BAG3 components of the CASA complex and the autophagic receptor p62 are important for mitotic cell rounding, the process by which actin retraction fibers are formed.

At the end of the cell division, the actin fibers, along with myosin, form the contractile ring, an important structure that, during cytokinesis, allows the abscission of the two daughter cells without leaks in the cytoplasm. Recently, it has been reported that in HeLa cells, HSPB8 silencing does not affect the assembly of the contractile ring, even if the cells resulted abnormally enriched in actin fibers, but HSPB8 silencing resulted in actin fiber disassembly during cytokinesis [79]. HSPB8 silencing also causes a delay in cytokinesis, and the daughter cells remain connected via a long disorganized intercellular actinic bridge due to an abnormal actin fibers accumulation. The cytokinesis failure could be explained by the action carried out by the HSPB8-BAG3 complex in the autophagic clearance of components of actin-based cytokinetic structures; in fact, the induction of autophagy can counteract the effects of HSPB8 silencing on actin fibers accumulation.

Collectively, these observations indicate that the HSPB8-BAG3 components of the CASA complex play an important role in quality control from the early stages of mitosis to the final part of cytokinesis.

## 7. Role of HSPB8 in Inflammation

The expression and role of HSPB8 were deeply investigated in different types of cells activated during inflammation (Figure 2). Roelofs and colleagues analyzed the involvement of HSPB8 in the pathogenesis of rheumatoid arthritis (RA), showing that HSPB8 could induce monocyte-derived dendritic cell maturation and cytokine production in a TLR4-dependant way. At the same time, HSPB8 was found to be highly expressed in synovial tissues from RA patients, and the release of HSPB8 in the synovial joint could potentially lead to an ongoing activation of inflammatory cells, thereby amplifying the inflammatory loop of synovial inflammation [80].

HSPB8 also is able to induce an intense interleukin-6 secretion in cultured pericytes and astrocytes [81], an aspect that may be related to neuroinflammation. Neuroinflammation is a hallmark of different neurodegenerative diseases, and HSPB8 upregulation in astrocytes is prominent in many neurological diseases, such as multiple sclerosis (MS), Alzheimer disease (AD), Parkinson’s disease (PD), and X-linked adrenoleucodystrophy (X-ALD), in which it associates with astrocyte reactivity. In Alzheimer disease (AD), in which the role of different HSPBs was analyzed, it was demonstrated that HSPB8, HSPB6, and HSPB2 co-localize with cerebral amyloid angiopathy (CAA) in AD brains; notably, in human leptomeningeal smooth muscle cells and human brain astrocytes, HSPB8 increases the secretion of the inflammatory factors interleukin-8 (IL-8), soluble ICAM-1, and monocyte chemoattractant protein 1, suggesting that HSPB8, possibly working in conjunction with other HSPBs, can act as a mediator of the inflammatory reactions associated with CAA [82]. In MS, which is a chronic demyelination disease, HSPB1 and HSPB8 expression is enhanced in the active stages of demyelination in white matter lesions exclusively in astrocytes, and this is apparently a secondary response to damage; HSPB1 and HSPB8 activation has been proposed to play a role in controlling inflammation and promoting tissue repair [83].

In X-ALD, another genetic disorder in which demyelination occurs as a consequence of the accumulation of very long chain fatty acids, neuroinflammation is an important component of the disease. In this case, high HSPB8 expression was observed in astrocytes in X-ALD preactive lesions, demonstrating that several cells, especially astrocytes, are already stressed well before demyelination occurs [84]. Further, in the case of ALS, in which neuroinflammation apparently modulates disease progression [85], the association between disease duration, motor neuron damage, and expression of the stress-inducible HSPBs was proposed. In fact, in ALS astrocytes located in the lateral columns throughout the spinal cord, HSPB1, HSPB6, HSPB8, and HSP16.2 (*C. elegans* HSPB5 ortholog) expression is markedly increased. Moreover, a short disease duration is apparently correlated with less motor neuron loss, more microglial activity, and increased HSPB5 and HSPB8 expression, possibly because of a tight relationship in ALS between stressors (e.g., increased proteostasis and oxidative stress) and astrocyte reactivity, microglial activation, and survival [85].

The role of HSPB8 has not yet been analyzed in cancer inflammation. Inflammation and cancer often go hand in hand. On one hand, the tumor triggers an inflammatory response; on the other hand, the inflammatory context feeds the aggressiveness of the tumor and the dissemination of metastases. Interestingly, Colunga and colleagues, investigating in melanoma the anti-tumor activity of an oncolytic virus DPK in DPK-treated melanoma xenografts, observed an upregulation of HSPB8, Beclin-1, and pro-inflammatory cytokine TNF-α and the activation of caspase-1 and pyroptosis cell death-related protease [86]. Since a direct relationship between HSPB8 expression and inflammatory cell death was not investigated, further studies should be addressed to determine the role of HSPB8 in cancer inflammation.

## 8. Role of HSPB8 in Cancer Development and Progression

**Figure 4:**
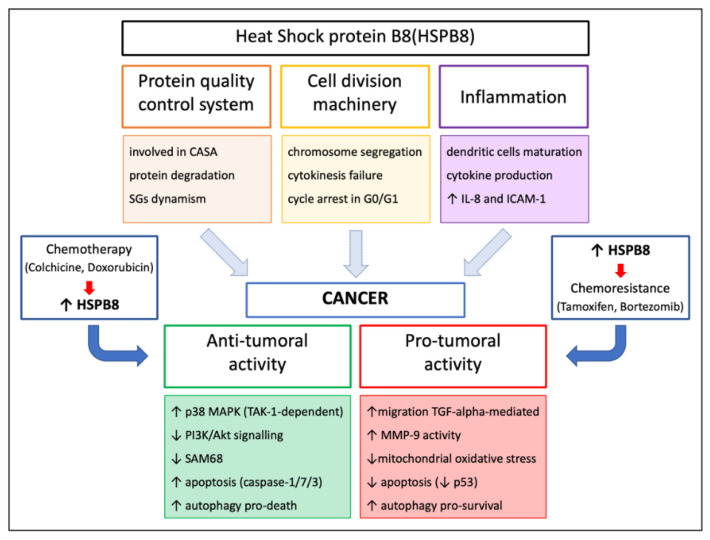
Schematic representation of HSPB8 roles in cancer development and progression.

In cancer cells, HSPB8 is differentially expressed and displays a dual and opposite role depending upon the type of tumor considered. In fact, in some tumors, HSPB8 promotes tumor growth (e.g., in BC [77,87], lung cancer [88], multiple myeloma [89], ovarian cancer [90], and gastric cancer [91,92]), while in other tumors (e.g., in melanoma [41,93,94], leukemia [39,40,92,95], glioblastoma [69,96], hepatocarcinoma [97,98,99], and prostate cancer (PC) [40,100,101,102]), HSPB8 has the opposite effect and reduces tumorigenesis (Figure 4). Here, we will briefly summarize how and when HSPB8 may act as a pro-cancer or anticancer agent.

### 8.1. Pro-Tumoral Activity of HSPB8

Several studies have shown that an abnormal expression of HSPB8 in cancer cells may be associated with tumor progression (Table 1). For example, the expression of the exogenous *Drosophila* small mitochondrial HSPB8 in human cancer cells increases the malignant properties of the cells and enhances the resistance to a variety of anticancer drugs; this process seems to correlate with an inactivation of the wild-type tumor suppressor protein p53 [103]. Furthermore, HSPB8 in melanoma cell lines and primary melanoma tissues is expressed at levels higher than normally found in melanocytes, and this could be related to the activation of growth-associated transcription factors, such as E2F and/or cyclin-dependent kinases (cdk), such as cdk4 [39].

HSPB8 has been identified as a potential key actor in the progression of human BC [87], where it appears to be generally overexpressed, particularly in estrogen receptor (ER)-positive (ER^+^) BC [104,105]. 17β-estradiol exposure has been shown to increase *HSPB8* mRNA [87,106] and protein levels in ER^+^ BC cells (MCF-7) [77,107], but, as expected, not in ER^−^ BC cells (MDA-MB-231). Thus, this estrogen-mediated induction of HSPB8 requires at least one isoform of the ERs, as it does not occur in ER^−^ cells, and this is confirmed by the fact that estrogen-dependent enhancement of HSPB8 expression is prevented by the pure ER antagonist ICI182.780 (faslodex, fulvestrant) [107]. Some studies have evidenced the role of autophagy in the development of tamoxifen resistance in BC and have reported a possible role of HSPB8 in mediating this chemoresistance. Indeed, tamoxifen induces autophagy in the tamoxifen-sensitive (tamS) cells but has no effect on those which were transformed in tamoxifen-resistant (tamR) cells; despite this, tamR cells are normally sensitive to the induction of autophagy if this is promoted by other pro-autophagic drugs. The fact that HSPB8 is expressed at higher levels in tamR cells than in those still sensitive to this anti-estrogen suggests that this chaperone may be involved in the acquisition of chemoresistance to the drug [108]. In fact, HSPB8 silencing in tamR MCF-7 cells reduces their proliferation and, surprisingly enough, this effect is enhanced in the presence of tamoxifen, which is not expected to exert any role in the tumor properties of these cells. On the contrary, the ectopic expression of HSPB8 in tamS cells allowed them to grow also in the presence of tamoxifen [108]. Furthermore, HSPB8 silencing in tamR cells correlates with autophagy induction, while the ectopic expression of HSPB8 in tamS cells reduces the percentage of autophagic cells compared to the control cells [108].

A recent study from our group has demonstrated that HSPB8 silencing correlated with an increased number of cells resting in the G0/G1 phase, reducing the ability of the cells to pass through the restriction point, causing a robust decrease in MCF-7 cell proliferation [77]. Other reports [87,109,110] suggested that HSPB8 mediates the cyclin D1 effects on radiation sensitivity, acting as a cdk-independent target of cyclin D1. Notably, cyclin D1 can regulate HSPB8 levels in a ligand-independent and steroid coactivator-dependent manner [87]. In addition, HSPB8 levels are tightly correlated with cyclin D1 and ER status in BC specimens, and HSPB8 enhances the sensitivity to radiation. Thus, radiation therapy might be better delivered to ER^+^ and cyclin D1-positive BCs along with estradiol itself, rather than in combination with anti-estrogenic agents [109]. Furthermore, HSPB8 expression is markedly elevated in radioresistant BC lines compared to parental MCF-7 and MDA-MB-231 cells, revealing the reprogramming of the heat shock proteome during the development of radioresistance in BC [111].

Notably, HSPB8 also modulates cell migration. Indeed, the pro-migratory effect induced by 17β-estradiol treatments on MCF-7 cells was abolished by HSPB8 silencing [77]. The pro-migratory activity of HSPB8 in cancer cell migration has also been described in a serous ovarian cancer cell line (SKOV3.ip1), in which overexpressed HSPB8 was found to increase TGF-α-induced ovarian cell migration [90]. As expected, HSPB8-silenced SKOV3.ip1 cells showed a significant reduction in TGF-α-induced migration compared with that observed in the control cells [90].

In accordance with that observed in breast and ovarian cancer, HSPB8 expression in lung adenocarcinoma (LUAC) was found to be higher in tumor tissue than in normal lung tissues, while HSPB8 overexpression enhanced cancer cell proliferation and migration; instead, the opposite effect was found after HSPB8 silencing. HSPB8 exerts this effect, inhibiting mitochondrial oxidative stress and increasing mitochondrial function [88].

In gastric cancer, the evaluation of the expression levels of HSPB8 may help to identify high-risk gastric cancer patients and thus aid the selection of appropriate therapies [92]. In fact, the survival times of gastric cancer patients with high HSPB8 expression are significantly shorter than those with low HSPB8 expression, suggesting that HSPB8 plays an important role in tumor prognosis; on these bases, it is also expected that HSPB8 could be a potential target for the prevention and treatment of gastric cancer.

The degradation of the extracellular matrix (ECM) is a signal for the beginning of invasion and metastasis, and matrix metallopeptidases (MMPs) are important factors involved in ECM degradation during invasion and metastasis [112]. Notably, cancer MMP-9 significantly correlates with depth of invasion and lymph node metastasis. Moreover, MMP-9-positive gastric cancer patients have worse outcomes than those with MMP-9-negative tumors [91]; of note, MMP-9 silencing results in the inhibition of cell growth and invasion of SGC7901 gastric cancer cells in vitro and in vivo [113]. There is generally a tight correlation between HSPB8 and MMP-9 expression, with higher invasive and metastasizing activity in HSPB8 high-expression cancer cells [92]. In addition, HSPB8 was highly expressed in depth of invasion, especially in T3 and T4 carcinomas, while patients with lymph node metastasis tend to show elevated HSPB8 expression.

Myeloma research has linked HSPB8 levels and resistance to proteasome inhibitors. Proteasome inhibitors are the preferred therapy for myeloma and the main drug used is bortezomib (Velcade). The proteasome is a complex that degrades the ubiquitinated intracellular proteins, leading to their elimination and therefore playing an essential role in maintaining normal cellular functions. In tumor cells, the proteasome plays an essential role in controlling growth, angiogenesis, and cell apoptosis. In fact, proteasome inhibition leads to the intracellular accumulation of misfolding proteins, causing proteotoxic stresses on the endoplasmic reticulum and consequently a caspase-4- and -12-dependent apoptosis, triggered by CHOP. Velcade alters the balance between protein synthesis and degradation, leading to cell death by apoptosis, but the treatment is effective in many, but not all, patients. It has been demonstrated that Velcade-resistant myeloma cells showed a reduction in ubiquitinated misfolded proteins despite the inhibition of the proteasome; in fact, these resistant cells expressed high levels of HSPB8, and this resulted in enhanced autophagic flux and lysosomal degradation of protein aggregates. Therefore, the HSPB8-induced autophagic removal of misfolded proteins is one of the causes of resistance to Velcade. Hence, high levels of HSPB8 in myeloma represent a factor of cell survival and resistance to therapy [89].

### 8.2. Anti-Tumoral Activity of HSPB8

In the previous section, we described how in many tumors the expression of HSPB8 correlates with the degree of tumorigenesis (Table 2). However, in several cases, even aggressive tumors are characterized by weak or no expression of HSPB8 [114]. Indeed, the first studies on the role of HSPB8 in cancer were conducted in melanoma and were highly enigmatic. In fact, HSPB8 is highly expressed in melanoma cells and in primary melanoma tissues compared to melanocytes and its overexpression represents a marker of cellular transformation [39]. At the same time, HSPB8 expression is regulated by the methylation status of its promoter both in melanoma and other cancers, such as prostate cancer [115], and demethylating agents (such as 5-Aza-deoxy-cytidine) induce HSPB8 expression, together with an apoptotic response mediated by caspase-3 activation [40]. In addition, in human melanoma A2058 and SKMEL2 cells, an inducible overexpression of HSPB8 causes DNA fragmentation due to the activation of the intrinsic apoptotic pathway mediated by caspase-9 and -3 activation [93]; HSPB8 also recruits TAK1, inducing its activation with consequent involvement of the p38-MAPK pathway. This effect is not present in the same cell type expressing a mutated version (W51C) of HSPB8 which exerted an anti-apoptotic activity. The active phosphorylated form of TAK-1 (pTAK-1) phosphorylates β-catenin and promotes its ubiquitination and subsequent proteasomal degradation. This event inhibits cdk22 activity, inducing cell cycle arrest in the G2 stage [93]. In addition, in A2058 and A375 melanoma cell lines, HSPB8 overexpression induced by 5-Aza-deoxy-cytidine treatment triggers apoptotic cell death, which correlates with the levels of HSPB8 DNA methylation. For this reason, HSPB8 has been proposed as a possible molecular marker for demethylated therapies [41]. Noteworthy is that, in human melanocytes, HSPB8 causes cell cycle arrest, while HSPB8 is silenced by aberrant DNA methylation in the majority of human melanomas. In parallel, HSPB8 upregulates Beclin-1 expression in A2058 xenografts through mTOR phosphorylation, causing Beclin-1-mediated caspase-1 cleavage [94].

HSPB8 is expressed at low levels also in a hematologic tumor cell line and bone marrow samples from patients with leukemia [40,41,95]. This is apparently due to the presence of a CpG island at 216 bp upstream of the putative HSPB8 transcription start site [40]. In fact, 5-Aza-deoxy-cytidine treatment increases HSPB8 expression in a dose-dependent manner and reduces colony formation of K62 and Namalwa cells, blocking cellular proliferation in vitro and in vivo. Thus, HSPB8 expression levels can be seen as a biomarker in following the use of 5-Aza-cytidine in leukemias and lymphoma [95].

High levels of *HSPB8* mRNA were observed in U87 glioblastoma cells [69], in which the HSPB8 protein inhibits Sam68 (also named GAP-associated tyrosine phosphoprotein p62) [69]. Sam68 exerts an important role in cell signal transduction, transcription, RNA metabolism, proliferation, and the survival of cancer cells [116,117,118]; thus, HSPB8 expression might be inversely correlated with the action of Sam68 and the ratio of HSPB8/Sam68 could be indicative of the proliferative potential of glioblastoma [96].

Hepatocellular carcinoma (HCC) is the most common form of neoplasm and corresponds to 85–90% of primary liver tumors. When the levels of HSPB8 were analyzed in tumor tissues of patients with HCC, no differences were observed in comparison to non-tumor tissues [97]. In fact, HSPB8 is both expressed in human HCC tumors and the adjacent non-tumor liver tissues. However, it has been shown that a group of patients with low levels of the HSPB8 protein more frequently present moderately or poorly differentiated HCC, suggesting that HSPB8 plays a suppressive role in the progression of HCC [98]. Moreover, HSPB8 downregulation in a typical hepatoma cell line, HuH-7, caused a marked increase in cell migration induced by TGF alpha. The HSPB8 protein interacts with PI3K (as demonstrated by coimmunoprecipitation studies) to exert its inhibitory effect on cell migration and invasion, thus involving the PI3K/AKT pathway [98]. Of note, a prognosis model based on autophagy-related gene signatures in HCC has been recently postulated and HSPB8 results included among the biomarkers associated with the survival and clinical stage of HCC [99].

In PC, HSPB8 is expressed at low levels and silenced by methylation [40,114]. However, a recent study performed with integrated bioinformatics analysis showed that the expression levels of the transcription factor HOXB13 correlated with the aggressiveness of metastatic PC, and that HOXB13 directly regulates the expression of *CIT/STK21* and *HSPB8*. In particular, HOXB13 negatively regulates *HSPB8*; in fact, HOXB13 silencing or HSPB8 induction through colchicine treatment significantly reduces the migration of highly aggressive PC cells [100]. Notably, HOXB13 is a critical regulator of castration-resistant PC (CRPC), particularly in response to treatments with second-generation antiandrogens [101]. HOXB13 may also promote prostate tumorigenesis through direct transcriptional repression of HSPB8, confirming that metastatic PCs with low HSPB8 levels are hyperproliferative.

In addition, an autophagic-related gene expression signature was analyzed in PC in order to predict overall survival and disease-free survival in PC patients: 234 genes associated with autophagy were analyzed, and of these, 22 genes are associated with PC progression, including HSPB8. This result showed that HSPB8 may act as a promising prognostic molecular biomarker in PC [102].

Collectively, the knowledge on HSPB8 role in cancer demonstrates that this protein plays a different role related to the tumor context. To date, there are no clear explanations for this peculiar behavior. A suggestive hypothesis could be that the different roles of HSPB8 depend on its ability to form multiprotein complexes that modulate its chaperone activity. Indeed, as described above, HSPB8 activates CASA upon binding the co-chaperone BAG3. It is known that the autophagy activation in tumors is a complex and dual process that may lead to both cell death and cell survival [119], so the ability of HSPB8 to activate this process could explain its contradictory effects.

For these reasons, it could be important in the future to investigate how HSPB8 interacts with BAG3, as well as if different co-chaperones or other proteins may be involved in the modulation of HSPB8 activity in tumors. Thus, it will be fundamental to understand how this interaction can trigger selective autophagy (CMA or CASA) affecting the fate of cancer cells.

## 9. Modulation of HSPB8 Expression

As detailed above, HSPB8 has a controversial dual role in cancer. Therefore, both HSPB8 inducers and repressors could be of interest against cancer, depending upon the specific tumor to be treated.

Estrogens and selective estrogen receptor modulators (SERMs) are the first molecules identified as HSPB8 inducers [77,87,106,107,120]. Some SERMs, such as tamoxifen and 3β-diol (which is an endogenous androgenic derivative characterized by a potent estrogenic activity mediated by ERbeta [121]) induce HSPB8 expression in MCF-7 cells [77], while other SERMs, such as raloxifen and genistein, are not effective [77]. Notably, tamoxifen induces apoptosis and autophagy in BC cells [122], and it is largely used in BC treatment [123]. Unfortunately, BC cells often become resistant to tamoxifen, giving rise to more aggressive tumors. As mentioned before, HSPB8 appears to be directly involved in the acquisition of tamoxifen resistance [108,120]. HSPB8-related effects seem to be mediated by the kinase LMTK3, an upstream regulator of HSPB8 expression, that was identified as a mediator of tamoxifen resistance in BT-474 BC cells [124]. Since these adverse HSPB8 effects in tamR cells prevent patients from benefiting from tamoxifen treatment, an alternative approach has been based on AZD8055, a mTOR kinase inhibitor, which downregulates HSPB8 expression in tamR BC cells. This treatment decreases HSPB8 levels and positively correlates with reduced cell proliferation [120], indicating that AZD8055 is promising for the therapy of tamR BC tumors. Further, the anti-inflammatory statin atorvastatin (ATV) was identified as a negative modulator of HSPB8, both in in vitro and in vivo atherosclerotic models [125,126], but so far has not been tested in BC.

Not only estrogens, but also the other female steroid hormones, progestins, have been identified as HSPB8 inducers. In a gene expression profiling analysis performed on T47D BC cells treated with the synthetic progestin R5020, HSPB8 was found to be upregulated, particularly during the G2/M phase [127]. Notably, progestin-mediated *HSPB8* gene transcription is regulated by a complex containing SP1, cyclin D1, and phospho-Ser345 PR, which binds with REs and SP1 DNA-binding motifs located in the promoter region of the *HSPB8* gene [127].

Other potent HSPB8 inducers are several proteasome inhibitors [128], some of which are currently used as therapeutic options against cancer, due to their ability to activate autophagy [129]. In fact, when the ubiquitin-proteasome pathway is inhibited, cells upregulate autophagic proteins, including those of the CASA complex. Specifically, the proteasome inhibitors MG132, Velcade, and lactacystin upregulate HSPB8 and its co-chaperone BAG3, both at transcriptional and protein levels, in different cell types [20,56,128,130]. As mentioned above, Velcade is used for the treatment of different types of tumors, but, unfortunately, cancer cells may develop drug resistance. Similar to the case of tamoxifen, in Velcade acquired resistance, HSPB8 plays a crucial role [89]. For example, in myeloma cells, HSPB8 is robustly overexpressed in Velcade-resistant cells, where it confers a selective resistance to the drug [89].

The fact that HSPB8 expression increases when cells preferentially activate autophagy correlates with data showing that autophagy inducers may upregulate HSPB8 expression. An example is the disaccharide trehalose that activates autophagy via the lysosomal-mediated TFEB pathway [131,132]. By activating autophagy, trehalose assists the removal of several toxic misfolded proteins [131,133,134,135], and, not surprisingly, trehalose has been proven as a therapeutic approach in in vitro and in vivo cancer models [136,137,138]. Of note, still related to PQC modulation, HSPB8 and BAG3 expression has been found to respond to some proteotoxic stress via the activation of the NF-κB transcription factor [22,73]. It is known that NF-κB regulates the expression of a hundred genes, involved in inflammation, immunity, proliferation, and cell death; thus, it is difficult to foresee how and if the modulation of HSPB8 expression via NF-κB may be therapeutically useful.

Other HSPB8 inducers are the compounds able to mount the stress response mediated by the activation of HSF1, the key master regulator of HSP family gene expression. Among these are the HSPs inducer geranylgeranylacetone (GGA) [139,140] and the potent HSPs inducer multitarget small molecule *N*-((5-(3-(1-benzylpiperidin-4-yl)propoxy)-1-methyl-1H-indol-2-yl)methyl)-*N*-methylprop-2-yn-1-amine (ASS234) [141]. Both molecules have been successfully tested on AD models [142,143,144,145,146,147], exerting a protective role mediated by HSPs in general, and especially by HSPB8. Interestingly, GGA has been proven to induce apoptosis in some tumors, including melanoma [148,149], and suggested as a potential therapeutic agent for these tumors. Recalling the HSPB8 pro-apoptotic activity in melanoma cells, it would be interesting to investigate if HSPB8 may be involved in the effects observed after GGA treatment.

Using a high-throughput screening approach to find small molecules for the treatment of misfolded protein diseases, we identified two other drugs capable of positively modulating HSPB8 gene expression: colchicine and doxorubicin [24]. The two molecules both increased HSPB8 expression in a neuroblastoma ALS cell model, reducing the accumulation of toxic misfolded proteins. Colchicine is a well-known microtubule destabilizing agent, used for the treatment of gout, Mediterranean fever, Bechet’s disease, and recurrent pericarditis, and is now under investigation in a phase II clinical trial for ALS [150]. Moreover, colchicine is widely used as a research tool for the study of microtubule dynamics. Microtubules are an important target for anticancer agents and drugs that disrupt their assembly/disassembly are widely used in chemotherapy. Unfortunately, colchicine is poorly used in clinics because of its toxicity and the development of multi-drug resistance, but several drugs targeting the colchicine-binding site have recently been developed and under trials [151]. Further, the anthracycline antibiotic doxorubicin is known for its ability to kill cancer cells and commonly used to treat many forms of cancer, including BC [152]. Furthermore, in the case of doxorubicin, cancer cells may develop drug resistance [153]. Considering the negative role exerted by HSPB8 in BC cells, we can postulate that HSPB8 may play a role in acquiring resistance, and therefore this aspect in doxorubicin treatment should be carefully considered.

## 10. Conclusions

All data reported in this review suggest that HSPB8 is deeply involved in the modulation of the appearance and the progression of many types of cancer in humans. However, as extensively discussed, HSPB8 activity can be either beneficial or detrimental for cancer cell growth, migration, and death. These aspects must be taken into account when therapeutic approaches aimed to up- or downregulate HSPB8 expression are tested and their use should be carefully tailored for each specific form and stage of tumor. Furthermore, it must be considered if these tumors have been previously treated with chemotherapeutic agents that have impacted on HSPB8 expression, allowing them to develop resistant cancer cell clones. Since HSPB8 mainly exerts action in PQC by activating CASA, a selective form of autophagy devoted to route misfolded proteins to the autophagosomes for lysosomal degradation and triggered by several stressful events, it may be possible that the adverse effects exerted by HSPB8 on tumor resistance are related to enhanced stress tolerance, allowing cells to escape from the cytotoxic properties of the chemotherapy. The hypothesis that many of the HSPB8 effects in tumors are due to its ability to facilitate autophagy (CASA) may also help to explain its controversial effects in different tumors, since CASA could also be indirectly modulated by other specific chaperones, co-chaperones, and ubiquitin ligases involved in the PQC system. Moreover, many other possible interactors may selectively potentiate or reduce CASA or route protein clearance to alternative degradative systems (e.g., the proteasome or the CMA). In fact, autophagy in cancer is a complex phenomenon characterized by both pro-death and pro-survival opposite effects but also tightly interconnected with the function of the proteasome. Unfortunately, still little is known about the ways in which HSPB8 can activate CASA in tumors and whether HSPB8 exerts its action by interacting with cell-specific proteins. Beside this main role, HSPB8 is also involved in other important cellular processes, such as the control of cell division, the inflammatory response, survival, and cell death. Due to the complexity of these synergic or antagonistic activities of HSPB8, and despite numerous studies, the role of HSPB8 in tumors is still extremely enigmatic.

To date, the regulation of human HSPB8 expression could represent an important event in the tumorigenesis process. Therefore, it is important to deeply investigate the molecular mechanisms underlying human HSPB8 activities in order to evaluate in which type of cancer its induction/activation or downregulation/inactivation may represent a potential treatment.

## Figures and Tables

**Table 1:** HSPB8 pro-tumoral activity.

Tumor	Effects	Refs.
Breast cancer	Pro-proliferative	[77,87,104,105,106,107,109]
	Pro-migratory	[77]
	Drug resistance (tamoxifen)	[108]
Gastric cancer	Pro-proliferative	[92]
	Pro-invasive	[91,113]
Lung adenocarcinoma	Pro-proliferative	[88]
	Pro-migratory	[88]
Melanoma	Pro-proliferative	[39]
Myeloma	Pro-proliferative	[89]
	Induction of drug resistance	[89]
	Autophagy activation	[89]
Ovarian cancer	Pro-migratory	[90,110]

**Table 2:** HSPB8 anti-tumoral activity.

Tumor	Effects	Refs.
Glioblastoma	Anti-proliferative	[69,96]
Hepatocarcinoma	Anti-proliferative	[98]
	Anti-migratory	[98]
	Anti-invasive	[98]
Leukemia	Anti-proliferative	[95]
Melanoma	Anti-proliferative	[40,100,101]
	Pro-apoptotic	[40,100,101]
Prostate cancer	Anti-proliferative	[40,100,101]
	Anti-migratory	[40,100,101]
	Anti-invasive	[40,100,101]

## Abbreviations

ACD	alpha-crystallin domain
AD	Alzheimer disease
ALS	amyotrophic lateral sclerosis
ATV	anti-inflammatory statin atorvastatin
BAG3	Bcl2-associated athanogene 3
BC	breast cancer
CAA	cerebral amyloid angiopathy
CASA	chaperone-assisted selective autophagy
cdk	cyclin-dependent kinase
CMA	chaperone-mediated autophagy
CMT	Charcot–Marie–Tooth
CRYAC	alpha-crystallin C chain
CRPC	castration-resistant prostate cancer
CTD	C-terminal domain
DRiP	defective ribosomal product
ECM	extracellular matrix
E2IG1	E2-induced gene 1 protein
ER	estrogen receptor
FTD	frontotemporal dementia
GGA	geranylgeranylacetone
IL	interleukin
HCC	hepatocellular carcinoma
HD	Huntington’s diseases
dHMN	distal hereditary motor neuropathy
HSF	heat shock transcription factors
HSR	heat shock response
HSP	heat shock protein
sHSP	small heath shock protein
LUAC	lung adenocarcinoma
MMP	matrix metallopeptidase
MS	multiple sclerosis
MTOC	microtubule organization center
NF-κB	nuclear factor-Kappa B
NTD	N-terminal domain
PC	prostate cancer
PKA	protein kinase A
PKC	protein kinase C
PD	Parkinson’s disease
PQC	protein quality control
RA	rheumatoid arthritis
SAM68	Src-associated in mitosis of 68 kDa
SBMA	spinal and bulbar muscular atrophy
SERM	selective estrogen receptor modulator
SG	stress granule
TAK1	transforming growth factor-beta-activated kinase
tamR	tamoxifen-resistant
tamS	tamoxifen-sensitive
UPS	ubiquitin–proteasome system
X-ALD	X-linked adrenoleukodystrophy
